# Sex hormones and physical function among the Chinese oldest-old and centenarian women

**DOI:** 10.1186/s12967-022-03539-9

**Published:** 2022-07-28

**Authors:** Qiao Zhu, Ping Ping, Pei Zhang, Chaoxue Ning, Yali Zhao, Yao Yao, Xiubing Li, Shihui Fu

**Affiliations:** 1 Central Laboratory, Hainan Hospital of Chinese People’s Liberation Army General Hospital, Sanya, China; 2 Main Station of Drug Instrument Supervision and Inspection, Joint Logistic Support Force of Chinese People’s Liberation Army, Beijing, China; 3 School of Life Science, Beijing Institute of Technology, Beijing, China; 4 Center for the Study of Aging and Human Development and Geriatrics Division, Medical School of Duke University, Durham, NC USA; 5 Center for Healthy Aging and Development Studies, National School of Development, Peking University, Beijing, China; 6 Department of Urology Medicine, The Third Medical Centre of Chinese People’s Liberation Army General Hospital, Beijing, China; 7 Department of Cardiology, Hainan Hospital of Chinese People’s Liberation Army General Hospital, Sanya, China; 8 Department of Geriatric Cardiology, Chinese People’s Liberation Army General Hospital, Beijing, China

**Keywords:** Centenarians, Estradiol, Oldest-old, Physical function, Sex hormones

## Abstract

**Background:**

Physical independence is crucial for overall health in the elderly individuals. The life expectancy of women has been shown to be higher than that of men, which is also known as the “male–female health-survival paradox”. Sex hormones may be one of the explanations. However, the relationships between sex hormones and physical function remain unclear in the elderly females. This study was designed to explore these relationships among the Chinese oldest-old and centenarian women.

**Methods:**

Data from 1226 women were obtained from the China Hainan Centenarian Cohort Study. Home interviews, physical examinations and blood analyses were conducted using standardized procedures. Variables including age, Han ethnicity, illiteracy, smoker, drinker, estradiol (E2), testosterone (T), follicle-stimulating hormone, and luteinizing hormone were used in the multivariate logistic and linear regression analyses.

**Results:**

In all the participants, age [beta (95% confidence interval): − 0.84 (− 0.98, − 0.71)] and E2 levels [beta (95% confidence interval): − 0.22 (− 0.28, − 0.17)] were negatively associated with activities of daily living (ADLs) in the multivariate linear regression analyses (P < 0.05 for all). We also observed significantly negative associations of age [odds ratio (95% confidence interval): 0.90 (0.88, 0.91)] and E2 levels [odds ratio (95% confidence interval): 0.98 (0.98, 0.99)] with physical normality in the multivariate logistic regression analyses (P < 0.05 for all). Age and E2 levels gradually decreased with increases in the ADL quartiles across all the participants (P < 0.05 for all).

**Conclusions:**

This study demonstrated that E2 levels were negatively associated with physical function among the Chinese oldest-old and centenarian women.

## Background

Aging is characterized by the decline in the physiological regulatory functions with adverse effects on physical health and functional autonomy [1]. Decreased physical function, including musculoskeletal, neurological, circulatory, cognitive, and mental function, can lead to a fragile physical state, triggering functional dependence in the elderly individuals. Elderly individuals are more susceptible to the decline in the activities of daily living (ADLs), such as eating, self-care, and movement [2]. As the pace of global aging accelerates, achieving healthy aging has become a primary issue. Epidemiological data have shown that the life expectancy of women are higher than those of men, which is also known as the “male–female health-survival paradox” [3, 4]. Sex hormones may be one of the explanations. Both estradiol (E2) and testosterone (T) production are controlled by the hypothalamic-pituitary–gonadal axis, which stimulates sex steroid production by increasing the secretion of gonadotropin-releasing hormone in the hypothalamus, which in turn stimulates the anterior pituitary to secrete gonadotropins, including luteinizing hormone (LH) and follicle-stimulating hormone (FSH). Loss of negative feedback by E2 and T on gonadotropin production after menopause results in an increase in the serum LH and FSH concentrations [5]. It is has been suggested that E2 may be associated with inflammatory processes, oxidative stress and lipid metabolism and beneficial to the cardiovascular system, physical function and healthy aging [6]. With the loss of E2, postmenopausal women undergo a number of processes in which risk factors for cardiovascular disease rapidly rise. However, age-related changes in the E2 and T levels in the older adults, especially older women, have not been fully explored. A previous analysis of women aged 18–75 years showed a steep decline in the T levels over early reproductive ages, flattened out in the midlife, and increased slightly in the later years [7]. The Baltimore Longitudinal Study of Aging reported that T declined with age before menopause and increased slightly afterward [8]. Given all of this information, little is known about the relationships between sex hormones and ADLs in the elderly females. Based on data from 1226 women over 80 years of age from the China Hainan Centenarian Cohort Study (CHCCS), we described age-related trajectories of sex hormones among the oldest-old and centenarian women, with a particular focus on their relationships with physical function.

## Methods

### Study population

The entire study sample was obtained from the CHCCS, which was conducted from June 2014 to December 2016. Based on a list of oldest-old and centenarian adults provided by the Department of Civil Affairs of Hainan Province, China, this cross-sectional study conducted a household survey with all the oldest-old and centenarian adults residing in the 16 cities and counties in the Hainan Province. In brief, a total of 1308 women (486 aged 80–99 years and 822 aged 100 years and over) were interviewed, with basic information and blood analyses available for 1226 participants. Home interviews, physical examinations and blood analyses were conducted using standardized procedures [9]. No participant was receiving sex hormone drugs or postmenopausal sex hormone replacement treatment.

### Functional performance

We used the Barthel index to evaluate ADLs in the elderly individuals. The Barthel index consists of ten items measuring a person’s daily activities: grooming, feeding, dressing, bathing, toilet use, transferring from bed to chair, walking, stair climbing, bowel continence, and urinary continence. Each item of ADLs is rated on a scale with a given number of points assigned to each level of activity, and the total scores range from 0 to 100 points with 5-point increments. The surveyors, who were strictly trained before the investigation, evaluated the Barthel index by observing the completion of each action. Higher Barthel index indicated better ADLs. Physical normality was defined by a total score ≥ 95 points, and physical decline was defined by a total score < 95 points [10].

### Hormone assays

A disposable vacuum negative pressure blood sampler was used by experienced nurses to collect blood samples from the elbow vein of the participants in a sitting position. Blood samples were stored at 4 °C and transported to our Central Laboratory within the 4 h. Serum E2 (pmol/L), T (nmol/L), FSH (mIU/mL) and LH (mIU/mL) levels were measured by electrochemiluminescence (Cobas E602) using the Elecsys Estradiol III Kit, Elecsys Testosterone II Kit, Elecsys FSH Kit, and Elecsys LH Kit (Roche, Germany). The lower detection thresholds were 18.4 pmol/L, 0.087 nmol/L, 0.100 mIU/mL, and 0.100 mIU/mL, respectively.

### Concomitant variables

Home interviews were conducted by native doctors and nurses who were trained to interview and could speak the local dialect to collect demographic details (age, ethnicity, and education) and lifestyle factors (smoker or nonsmoker and drinker or nondrinker) of the participants as concomitant variables. Ethnicity was divided into “Han ethnicity” and “non-Han ethnicity”. Education was classified as “illiteracy” and “literacy”.

### Statistical analyses

Data were analyzed using the Statistical Package for Social Sciences Version 17 (Chicago, IL, USA). Numbers and percentages are presented for categorical variables, and medians and interquartile ranges are presented for continuous variables with skewed distributions. Categorical variables were compared with Chi-square tests, and continuous variables with skewed distributions were compared with Mann–Whitney U tests. Multivariate linear regression analyses were used to explore independent factors associated with age and ADLs. Multivariate logistic regression analyses were used to determine independent associations with centenarian women/oldest-old women and physical normality/decline. Variables including age, Han ethnicity, illiteracy, smoker, drinker, and E2, T, E2/T, FSH, and LH levels were used in the multivariate logistic and linear regression analyses. A P < 0.05 indicated statistical significance.

## Results

A comparison of characteristics between oldest-old women and centenarian women is shown in the Table 1. Centenarian women accounted for 61.8% of the sample (758 participants). Centenarian women had significantly higher age, higher levels of E2, and lower levels of ADLs than oldest-old women (*P* < 0.05 for all). As shown in the Table 2, we observed that E2 levels [beta (95% confidence interval): 0.05 (0.03, 0.07)] were positively associated with age, whereas ADLs [beta (95% confidence interval): − 0.13 (− 0.15, − 0.11)] were negatively associated with age in the multivariate linear regression analyses (P < 0.05 for all). We then tested our results in the multivariate logistic regression analysis: a positive association between E2 levels [odds ratio (95% confidence interval): 1.04 (1.02, 1.05)] and centenarians and a negative association between ADLs [odds ratio (95% confidence interval): 0.94 (0.93, 0.95)] and centenarians remained significant in all the participants (P < 0.05 for all; Table 2). E2 levels gradually increased and ADLs gradually decreased with increases in the age quartiles (P < 0.05 for all; Table 3).

**Table 1:** Comparison of characteristics between oldest-old women and centenarian women in all the participants

Variables	Oldest-old women (N = 468)	Centenarian women (N = 758)	All (N = 1226)	P
Age (year)^a^	84.0 (82.0, 89.0)	102.0 (101.0, 104.0)	100.5 (87.0, 103.0)	< 0.001
Han ethnicity, n (%)^b^	414 (88.5)	672 (88.7)	1086 (88.6)	0.918
Illiteracy, n (%)^b^	432 (92.3)	732 (96.6)	1164 (94.9)	0.001
Smoker, n (%)^b^	13 (2.8)	52 (6.9)	65 (5.3)	0.002
Drinker, n (%)^b^	80 (17.1)	103 (13.6)	183 (14.9)	0.094
E2 (pmol/L)^a^	18.4 (18.4, 36.9)	33.2 (18.4, 56.1)	22.9 (18.4, 38.6)	< 0.001
T (nmol/L)^a^	0.4 (0.2, 0.9)	0.4 (0.2, 0.7)	0.4 (0.2, 0.8)	0.100
E2/T^a^	55.8 (23.9, 141.5)	95.6 (48.7, 204.4)	80.0 (37.1, 204.2)	< 0.001
FSH (mIU/mL)^a^	82.3 (65.9, 100.2)	81.4 (61.0, 101.0)	82.3 (62.7, 100.4)	0.211
LH (mIU/mL)^a^	36.3 (28.4, 42.7)	36.2 (28.4, 47.1)	36.3 (28.4, 45.7)	0.193
ADL^a^	100.0 (91.0, 100.0)	85.0 (60.0, 95.0)	90.0 (70.0, 100.0)	< 0.001

*ADL* activity of daily living; *E2* estradiol; *E2/T* estradiol/testosterone; *FSH* follicle-stimulating hormone; *LH* luteinizing hormone; *T* testosterone

^a^Continuous variables with skewed distributions were presented with medians (interquartile ranges) and compared with Mann–Whitney U tests

^b^Categorical variables were presented with numbers (percentages) and compared with Chi-square tests

**Table 2:** Multivariate linear regression analysis with age and multivariate logistic regression analysis with centenarian women/oldest-old women in all the participants

Variables	Age^a^		Centenarian women/oldest-old women^b^	
	Beta (95% confidence interval)	P	Odds ratio (95% confidence interval)	P
Non-Han ethnicity, n (%)	− 0.17 (− 1.68, 1.34)	0.823	0.78 (0.50, 1.22)	0.272
Literacy, n (%)	− 4.59 (− 6.70, − 2.48)	< 0.001	0.35 (0.18, 0.66)	0.001
Nonsmoker, n (%)	− 3.17 (− 5.28, − 1.07)	0.003	0.39 (0.19, 0.78)	0.008
Nondrinker, n (%)	1.08 (− 0.28, 2.43)	0.119	1.50 (1.01, 2.23)	0.047
E2 (pmol/L)^c^	0.05 (0.03, 0.07)	< 0.001	1.04 (1.02, 1.05)	< 0.001
T (nmol/L)^c^	− 0.29 (− 0.60, 0.01)	0.061	0.87 (0.80, 0.95)	0.002
E2/T^c^	0.00 (0.00, 0.01)	0.092	1.00 (1.00, 1.00)	0.055
FSH (mIU/mL)^c^	− 0.01 (− 0.04, 0.01)	0.266	1.00 (0.99, 1.00)	0.252
LH (mIU/mL)^c^	0.04 (− 0.01, 0.08)	0.148	1.01 (0.99, 1.02)	0.416
ADL^c^	− 0.13 (− 0.15, − 0.11)	< 0.001	0.94 (0.93, 0.95)	< 0.001

*ADL* activity of daily living; *E2* estradiol; *E2/T* estradiol/testosterone; *FSH* follicle-stimulating hormone; *LH* luteinizing hormone; *T* testosterone

^a^Multivariate linear regression analysis was used with non-Han ethnicity, literacy, nonsmoker, nondrinker, E2, T, E2/T, FSH, LH and ADL as independent variables and with age as dependent variable; ^b^Multivariate logistic regression analysis was used with non-Han ethnicity, literacy, nonsmoker, nondrinker, E2, T, E2/T, FSH, LH and ADL as independent variables and with centenarian women/oldest-old women as dependent variable

^c^Per 1 unit increase

**Table 3:** Multivariate logistic regression analyses with age quartiles and ADL quartiles in all the participants

Variables	Age quartiles^a^				ADL quartiles^b^			
	Age < 87 (n = 294)	87 ≤ Age < 100 (n = 319)	100 ≤ Age < 103 (n = 276)	Age ≥ 103 (n = 337)	ADL < 70 (n = 279)	70 ≤ ADL < 90 (n = 246)	90 ≤ ADL < 100 (n = 319)	ADL ≥ 100 (n = 382)
Age (year), n (%)^c^	–	–	–	–		**0.96 (0.93, 0.99)**^d^	**0.94 (0.92, 0.96)**^d^	**0.84 (0.82, 0.86)**^d^
Han ethnicity, n (%)	Ref.	1.34 (0.77, 2.31)	**2.09 (1.11, 3.94)**^e^	1.14 (0.65, 1.99)	Ref.	0.74 (0.43, 1.26)	1.04 (0.60, 1.83)	1.20 (0.65, 2.21)
Illiteracy, n (%)	Ref.	**3.81 (1.75, 8.27)**^e^	**2.65 (1.21, 5.79)**^e^	**4.08 (1.78, 9.34)**^e^	Ref.	0.93 (0.37, 2.31)	0.59 (0.26, 1.36)	1.23 (0.49, 3.06)
Smoker, n (%)	Ref.	0.91 (0.57, 1.46)	0.78 (0.46, 1.34)	0.67 (0.40, 1.12)	Ref.	0.83 (0.39, 1.80)	1.25 (0.61, 2.57)	1.23 (0.54, 2.79)
Drinker, n (%)	Ref.	1.48 (0.59, 3.77)	**3.10 (1.24, 7.73)**^e^	2.25 (0.91, 5.57)	Ref.	0.95 (0.57, 1.60)	0.79 (0.48, 1.32)	1.01 (0.60, 1.72)
E2 (pmol/L)^c^	Ref.	**1.02 (1.01, 1.04)**^d^	**1.03 (1.02, 1.04)**^d^	**1.03 (1.02, 1.04)**^d^	Ref.	**0.99 (0.99, 1.00)**^e^	**0.98 (0.97, 0.98)**^d^	**0.98 (0.97, 0.99)**^d^
T (nmol/L)^c^	Ref.	0.96 (0.86, 1.07)	0.90 (0.79, 1.02)	0.92 (0.82, 1.04)	Ref.	1.00 (0.90, 1.11)	0.94 (0.84, 1.06)	0.95 (0.85, 1.07)
E2/T^c^	Ref.	1.00 (1.00, 1.00)	**1.00 (1.00, 1.00)**^e^	**1.00 (1.00, 1.00)**^e^	Ref.	1.00 (1.00, 1.00)	1.00 (1.00, 1.00)	1.00 (1.00, 1.00)
FSH (mIU/mL)^c^	Ref.	0.99 (0.99, 1.00)	0.99 (0.98, 1.00)	1.00 (0.99, 1.01)	Ref.	1.00 (0.99, 1.00)	1.00 (0.99, 1.00)	1.00 (0.99, 1.01)
LH (mIU/mL)^c^	Ref.	1.02 (1.00, 1.03)	1.02 (1.00, 1.04)	1.01 (0.99, 1.03)	Ref.	1.00 (0.99, 1.02)	0.99 (0.97, 1.01)	**0.98 (0.96, 1.00)**^e^
ADL^c^	Ref.	**0.95 (0.94, 0.97)**^d^	**0.93 (0.92, 0.95)**^d^	**0.93 (0.92, 0.95)**^d^	–	–	–	–

Bold indicates statistical significance

*ADL* activity of daily living; *E2* estradiol; *E2/T* estradiol/testosterone; *FSH* follicle-stimulating hormone; *LH* luteinizing hormone; *T* testosterone

^a^Multivariate logistic regression analyses were used with age, ethnicity, education, smoker, drinker, E2, T, E2/T, FSH, LH and ADL as independent variables and with age quartiles as dependent variable

^b^Multivariate logistic regression analyses were used with age, ethnicity, education, smoker, drinker, E2, T, E2/T, FSH, LH and ADL as independent variables and with ADL quartiles as dependent variable

^c^Per 1 unit increase

^d^Significance at the level of < 0.001 (2-tailed)

^e^Significance at the level of < 0.05 (2-tailed)

Table 4 summarizes the characteristics by functional performance in all the participants. The proportion of physical normality was 44.8% (549 participants). The participants with physical normality had significantly lower age, lower levels of E2 and LH, and higher levels of ADLs than those with physical decline (P < 0.05 for all). As shown in the Table 5, age [beta (95% confidence interval): − 0.84 (− 0.98, − 0.71)] and E2 levels [beta (95% confidence interval): − 0.22 (− 0.28, − 0.17)] were negatively associated with ADLs in the multivariate linear regression analyses (P < 0.05 for all). We also observed significantly negative associations of age [odds ratio (95% confidence interval): 0.90 (0.88, 0.91)], E2 levels [odds ratio (95% confidence interval): 0.98 (0.98, 0.99)], and LH levels [odds ratio (95% confidence interval): 0.99 (0.97, 1.00)] with physical normality in the multivariate logistic regression analyses (P < 0.05 for all; Table 6). Age and E2 levels gradually decreased with increases in the ADL quartiles across all the participants (P < 0.05 for all; Table 3).

**Table 4:** Comparison of characteristics between participants with physical normality or decline in all the participants

Variables	Oldest-old women			Centenarian women			All		
	Physical decline^a^ (N = 117)	Physical normality^a^ (N = 351)	P	Physical decline^a^ (N = 560)	Physical normality^a^ (N = 198)	P	Physical decline^a^ (N = 677)	Physical normality^a^ (N = 549)	P
Age (year)^b^	87.0 (83.0, 91.0)	84.0 (82.0, 88.0)	< 0.001	102.0 (101.0, 104.0)	102.0 (101.0, 104.0)	0.637	102.0 (100.0, 104.0)	88.0 (83.0, 101.0)	< 0.001
Han ethnicity, n (%)^c^	97 (82.9)	317 (90.3)	0.030	494 (88.2)	178 (89.9)	0.521	591 (87.3)	495 (90.2)	0.117
Illiteracy, n (%)^c^	107 (91.5)	325 (92.6)	0.689	543 (97.0)	189 (95.5)	0.316	650 (96.0)	514 (93.6)	0.058
Smoker, n (%)^c^	4 (3.4)	9 (2.6)	0.871	37 (6.6)	15 (7.6)	0.643	41 (6.1)	24 (4.4)	0.191
Drinker, n (%)^c^	22 (18.8)	58 (16.5)	0.571	78 (13.9)	25 (12.6)	0.646	100 (14.8)	83 (15.1)	0.865
E2 (pmol/L)^b^	18.4 (18.4, 36.9)	18.4 (18.4, 36.9)	0.276	36.9 (18.4, 61.5)	18.5 (18.4, 38.7)	< 0.001	34.0 (18.4, 55.5)	18.4 (18.4, 36.9)	< 0.001
T (nmol/L)^b^	0.4 (0.2, 0.9)	0.4 (0.2, 0.9)	0.973	0.4 (0.2, 0.8)	0.3 (0.2, 0.6)	0.001	0.4 (0.2, 0.8)	0.4 (0.2, 0.8)	0.168
E2/T^b^	59.4 (25.7, 172.2)	55.8 (23.0, 141.5)	0.620	94.5 (48.4, 204.4)	102.2 (51.5, 204.4)	0.894	86.3 (42.7, 204.4)	68.1 (31.2, 184.2)	< 0.001
FSH (mIU/mL)^b^	83.1 (69.1, 103.2)	82.3 (64.0, 98.6)	0.122	82.3 (62.2, 102.2)	78.3 (59.2, 96.2)	0.035	82.3 (63.4, 102.3)	82.3 (61.8, 97.3)	0.084
LH (mIU/mL)^b^	38.1 (30.2, 46.2)	35.5 (27.5, 41.6)	0.002	37.5 (29.4, 47.8)	33.7 (26.2, 43.1)	0.001	37.5 (29.6, 47.6)	34.9 (26.9, 42.3)	< 0.001
ADL^b^	85 (70, 90)	100 (100, 100)	< 0.001	75 (50, 85)	95 (95, 100)	< 0.001	75.0 (55, 85)	100.0 (95, 100)	< 0.001

*ADL* activity of daily living; *E2* estradiol; *E2/T* estradiol/testosterone; *FSH* follicle-stimulating hormone; *LH* luteinizing hormone; *T* testosterone

^a^Physical normality was defined by a total score of ADL ≥ 95 points, and physical decline was defined by a total score of ADL < 95 points

^b^Continuous variables with skewed distributions were presented with medians (interquartile ranges) and compared with Mann–Whitney U tests

^c^Categorical variables were presented with numbers (percentages) and compared with Chi-square tests

**Table 5:** Multivariate linear regression analyses with ADL in all the participants

Models	Variables	Oldest-old women		Centenarian women		All	
		Beta (95% confidence interval)	P	Beta (95% confidence interval)	P	Beta (95% confidence interval)	P
Model 1^a^	E2 (pmol/L)^d^	− 0.03 (− 0.15, 0.10)	0.705	− 0.23 (− 0.29, − 0.17)	< 0.001	− 0.30 (− 0.35, − 0.24)	< 0.001
	T (nmol/L)^d^	− 0.01 (− 0.82, 0.81)	0.985	− 1.84 (− 3.16, − 0.53)	0.006	− 0.18 (− 0.99, 0.63)	0.667
	E2/T^d^	0.01 (− 0.01, 0.02)	0.527	0.00 (− 0.01, 0.01)	0.917	0.00 (− 0.01, 0.01)	0.761
	FSH (mIU/mL)^d^	0.00 (− 0.07, 0.07)	0.985	− 0.08 (− 0.16, 0.01)	0.096	− 0.05 (− 0.11, 0.02)	0.173
	LH (mIU/mL)^d^	− 0.11 (− 0.24, 0.03)	0.135	− 0.09 (− 0.26, 0.09)	0.317	− 0.12 (− 0.25, 0.01)	0.071
Model 2^b^	E2 (pmol/L)^d^	− 0.01 (− 0.14, 0.12)	0.932	− 0.23 (− 0.30, − 0.17)	< 0.001	− 0.30 (− 0.35, − 0.24)	< 0.001
	T (nmol/L)^d^	− 0.11 (− 0.95, 0.73)	0.797	− 1.85 (− 3.17, − 0.53)	0.006	− 0.14 (− 0.95, 0.67)	0.735
	E2/T^d^	0.00 (− 0.01, 0.02)	0.641	0.00 (− 0.01, 0.01)	0.871	0.00 (− 0.01, 0.01)	0.751
	FSH (mIU/mL)^d^	0.00 (− 0.07, 0.07)	0.971	− 0.08 (− 0.17, 0.01)	0.087	− 0.05 (− 0.11, 0.02)	0.167
	LH (mIU/mL)^d^	− 0.11 (− 0.24, 0.03)	0.139	− 0.09 (− 0.26, 0.09)	0.338	− 0.12 (− 0.25, 0.01)	0.069
Model 3^c^	E2 (pmol/L)^d^	− 0.01 (− 0.14, 0.12)	0.875	− 0.23 (− 0.30, − 0.17)	< 0.001	− 0.22 (− 0.28, − 0.17)	< 0.001
	T (nmol/L)^d^	− 0.04 (− 0.87,0.79)	0.924	− 1.83 (− 3.15, − 0.51)	0.007	− 0.37 (− 1.14, 0.40)	0.345
	E2/T^d^	0.01 (− 0.01, 0.02)	0.557	0.00 (− 0.01, 0.01)	0.869	0.00 (− 0.01, 0.01)	0.389
	FSH (mIU/mL)^d^	0.00 (− 0.07, 0.07)	0.947	− 0.08 (− 0.16, 0.01)	0.092	− 0.05 (− 0.11, 0.01)	0.095
	LH (mIU/mL)^d^	− 0.09 (− 0.23, 0.05)	0.210	− 0.09 (− 0.26, 0.09)	0.327	− 0.08 (− 0.20, 0.05)	0.218

*ADL* activity of daily living; *E2* estradiol; *E2/T* estradiol/testosterone; *FSH* follicle-stimulating hormone; *LH* luteinizing hormone; *T* testosterone

^a^Multivariate linear regression analyses were used with E2, T, E2/T, FSH and LH as independent variables and with ADL as dependent variable

^b^Multivariate linear regression analyses were used with ethnicity, education, smoker, drinker, E2, T, E2/T, FSH and LH as independent variables and with ADL as dependent variable

^c^Multivariate linear regression analyses were used with age, ethnicity, education, smoker, drinker, E2, T, E2/T, FSH and LH as independent variables and with ADL as dependent variable

^d^Per 1 unit increase

**Table 6:** Multivariate logistic regression analyses with physical normality^a^ in all the participants

Models	Variables	Oldest-old women		Centenarian women		All	
		Odds ratio (95% confidence interval)	P	Odds ratio (95% confidence interval)	P	Odds ratio (95% confidence interval)	P
Model 1^b^	E2 (pmol/L)^e^	1.00 (0.98, 1.02)	0.782	0.98 (0.97, 0.99)	< 0.001	0.97 (0.97, 0.98)	< 0.001
	T (nmol/L)^e^	1.00 (0.88, 1.14)	0.990	0.90 (0.78, 1.04)	0.158	1.02 (0.95, 1.11)	0.557
	E2/T^e^	1.00 (1.00, 1.00)	0.996	1.00 (1.00, 1.00)	0.881	1.00 (1.00, 1.00)	0.487
	FSH (mIU/mL)^e^	1.00 (0.99, 1.01)	0.826	1.00 (0.99, 1.01)	0.487	1.00 (0.99, 1.00)	0.649
	LH (mIU/mL)^e^	0.98 (0.96, 1.01)	0.136	0.98 (0.96, 1.00)	0.062	0.98 (0.97, 1.00)	0.006
Model 2^c^	E2 (pmol/L)^e^	1.00 (0.98, 1.02)	0.937	0.98 (0.97, 0.99)	< 0.001	0.97 (0.97, 0.98)	< 0.001
	T (nmol/L)^e^	0.98 (0.85, 1.12)	0.756	0.90 (0.78, 1.04)	0.168	1.03 (0.95, 1.11)	0.529
	E2/T^e^	1.00 (1.00, 1.00)	0.837	1.00 (1.00, 1.00)	0.846	1.00 (1.00, 1.00)	0.509
	FSH (mIU/mL)^e^	1.00 (0.99, 1.01)	0.831	1.00 (0.99, 1.01)	0.455	1.00 (0.99, 1.01)	0.635
	LH (mIU/mL)^e^	0.99 (0.96, 1.01)	0.185	0.98 (0.96, 1.00)	0.066	0.98 (0.97, 1.00)	0.006
Model 3^d^	E2 (pmol/L)^e^	1.00 (0.98, 1.02)	0.996	0.98 (0.97, 0.99)	< 0.001	0.98 (0.98, 0.99)	< 0.001
	T (nmol/L)^e^	0.99 (0.86, 1.13)	0.865	0.91 (0.79, 1.05)	0.180	0.99 (0.92, 1.08)	0.887
	E2/T^e^	1.00 (1.00, 1.00)	0.929	1.00 (1.00, 1.00)	0.835	1.00 (1.00, 1.00)	0.824
	FSH (mIU/mL)^e^	1.00 (0.99, 1.01)	0.823	1.00 (0.99, 1.01)	0.479	1.00 (0.99, 1.00)	0.448
	LH (mIU/mL)^e^	0.99 (0.97, 1.01)	0.267	0.98 (0.96, 1.00)	0.060	0.99 (0.97, 1.00)	0.031

*E2* estradiol; *E2/T* estradiol/testosterone; *FSH* follicle-stimulating hormone; *LH* luteinizing hormone; *T* testosterone

^a^Physical normality was defined by a total score of ADL ≥ 95 points, and physical decline was defined by a total score of ADL < 95 points

^b^Multivariate logistic regression analyses were used with E2, T, E2/T, FSH and LH as independent variables and with physical normality/decline as dependent variable

^c^Multivariate logistic regression analyses were used with ethnicity, education, smoker, drinker, E2, T, E2/T, FSH and LH as independent variables and with physical normality/decline as dependent variable

^d^Multivariate logistic regression analyses were used with age, ethnicity, education, smoker, drinker, E2, T, E2/T, FSH and LH as independent variables and with physical normality/decline as dependent variable

^e^Per 1 unit increase

When dividing all the participants into oldest-old women and centenarian women, we compared the characteristics between those with physical normality or decline in the Table 4. Among the oldest-old women, age and LH levels were significantly lower in the participants with physical normality than in those with physical decline (P < 0.05 for all). Among the centenarian women, E2, T and LH levels were significantly lower in the participants with physical normality than in those with physical decline (P < 0.05 for all). As shown in the Table 5, age [beta (95% confidence interval): − 0.51 (− 0.79, − 0.23)] was negatively associated with ADLs among the oldest-old women, and E2 [beta (95% confidence interval): − 0.23 (− 0.30, − 0.17)] and T [beta (95% confidence interval): − 1.83 (− 3.15, − 0.51)] levels were negatively associated with ADLs among the centenarian women in the multivariate linear regression analyses (P < 0.05 for all). In addition, the negative association between age [odds ratio (95% confidence interval): 0.91 (0.87, 0.96)] and physical normality remained robust among the oldest-old women but not among the centenarian women, and the negative association between E2 levels [odds ratio (95% confidence interval): 0.98 (0.97, 0.99)] and physical normality remained robust among the centenarian women but not among the oldest-old women in the multivariate logistic regression analyses (P < 0.05 for all; Table 6).

## Discussion

Physical independence is an important aspect of aging and a key factor affecting quality of life in the elderly population. Limited data exist on the relationships between sex hormones and physical function among the oldest-old and centenarian women in developing countries. Previous studies in China found a greater decline in physical function in the oldest-old group (≥ 80 years) than in the other two age groups (60–69 and 70–79 years) [11]. Studies in Spain showed that physical decline was independently associated with being a woman, lower educational status, and age over 85 years [12–15]. Studies in United States and Sweden showed that physical function remained steady over time, with a gradual tendency for performance to decline [16, 17]. A study conducted in Denmark suggested that the basis for improved physical function is superior living conditions and cognitive function in the older adults, as well as better aids in supporting mobility and independence [16]. Additionally, lifestyle, exercise, diet, and personality are also contributors that affect physical function [18, 19]. Our results showed a proportion of physical normality of approximately 44.8% and provide novel insight into this significant field of study, with large sample analyses of Chinese oldest-old and centenarian women. In this study, centenarian women were reported to have poor physical function than oldest-old women, and E2 levels were negatively associated with physical function among the Chinese oldest-old and centenarian women.

This finding may highlight the role of E2 in age-related loss of muscle mass and strength and provide the basis for E2-based interventions to prevent physical decline among those living past the age of one hundred. It is well known that skeletal muscle weakness is an adverse consequence of aging. Sarcopenia, an age-related loss of muscle mass and strength, is considered to be a precursor of frailty and can cause elderly individuals to lose physical independence [20]. The size and strength of women's skeletal muscle are affected by both age and ovarian hormones [21]. A meta-analysis of nearly 10,000 postmenopausal women showed that women who received sex hormone treatment had slightly stronger muscle strength than those not on sex hormone treatment [22]. It has been reported that E2 can not only affect muscle protein conversion and the ubiquitin–proteasome system but also protect skeletal muscle from apoptosis by acting on heat shock proteins and mitochondria [23–26]. Meanwhile, E2 is also produced in various brain regions [27]. E2 levels in the hippocampus are affected by E2 circulation. These results suggest that postmenopausal women may not be able to produce significant levels of E2 in both the reproductive tract and hippocampus [28]. Combined with the evidence that E2 levels in the hippocampus may have neuroprotective effects, decreased E2 levels in the postmenopausal women may make the hippocampus more vulnerable to degeneration [29]. Large randomized clinical trials have shown that sex hormone treatment did not improve cognitive function in the older postmenopausal women and may even have an adverse effect on cognitive function [30–33]. Brinton proposed a healthy cell bias of E2 action, suggesting that E2 could be beneficial to healthy cells but detrimental to degraded or already damaged cells [34]. This is because high doses of E2 increase intracellular Ca^2+^, which could exacerbate the development of neurodegeneration due to Ca^2+^ dysregulation [35]. A previous study identified a negative effect of E2 treatment on cognitive function, indicating a faster progression of Alzheimer's disease in the elderly individuals with E2 supplementation [36].

The physiological implications of changed sex hormones are still being clarified within the different age groups and with the different physical conditions. E2 treatment did not seem to have health benefits for elderly individuals with intact ovaries; elderly individuals who had their ovaries removed may experience harmful effects from E2 treatment [37]. Moreover, the effects of E2 in the brain regions involved in the response to stress and emotion may be associated with increased negative mood and depressive symptoms in the perimenopausal and postmenopausal women [38–40]. Studies have reported that E2 administration in the postmenopausal women without a history of mood disorders was associated with an increased negative mood response to a psychosocial stressor [41, 42]. This suggested a shift from beneficial effects of E2 in the premenopausal women to negative effects in the postmenopausal women without depression vulnerability. In other words, E2 has multidimensional effects on the body, cognition and emotion of postmenopausal women, which are different from those of premenopausal women. Our data showed that a significant association between E2 levels and physical normality remained in the centenarian women but not in the oldest-old women, prompting us to further explore these issues from multiple perspectives to elucidate the effects of E2 in women in different advanced age ranges. In our study, centenarian women had significantly higher levels of E2 and lower levels of ADLs than oldest-old women, providing E2 with the opportunity to influence physical function, whereas due to lower levels of E2 and higher levels of ADLs in the oldest-old women, E2 had no significant chance to affect physical function. It is possible that the different E2 levels and physical conditions within the different age groups resulted in the existence of a negative association in the centenarian women and not oldest-old women. A nationwide survey provided evidence that the prevalence of physical decline among Chinese older adults declined from 1997 to 2006, consistent with the Shanghai study from 1998 to 2008 [11, 43]. This may be because the fact that the new medical insurance policy implemented since 2000 may help older residents improve medical services, which to some extent explained that the oldest-old adults gained more benefits due to their age advantages at that time. On the other hand, this is relevant given that the improved living standard may help older residents improve their overall health status and physical function.

Our team reported that E2 levels of centenarian women were higher than those of oldest-old women. There are wide variations in the levels of sex hormones in women throughout the lifespan. The Baltimore Longitudinal Study of Aging reported progressive decreases in the E2 and T levels in the premenopausal women and increases at older ages [8]. Endogenous E2 and T were related to changed endothelial function in the postmenopausal women [44]. A 12-year follow-up study of postmenopausal women showed that abnormal E2 and T levels were associated with an increased risk for adverse events [45]. Meanwhile, LH and FSH are controlled by complex feedback loops in the hypothalamic-pituitary-ovarian axis. In the absence of changed feedback from sex hormones after menopause, increased LH levels are related to cognitive deficit and Alzheimer’s disease in the elderly individuals [46, 47]. Increased LH levels in patients with Alzheimer’s disease could exacerbate pathological cognitive decline [48–51]. This is because LH modulates amyloid-β protein precursor processing [52]. Elevated LH levels are correlated with increased plasma Aβ_1-40_ and Aβ_1-42_ levels [50]. Studies in patients without Alzheimer’s disease have shown that increased FSH levels improve cognitive function [53, 54]. In a study of 649 women over 70 years old without cognitive decline, it was found that higher FSH levels were associated with improved cognitive function [46, 55]. In our results, T, LH and FSH levels had no statistically significant associations with physical function among Chinese oldest-old and centenarian women.

This study has some potential limitations. First, we conducted a cross-sectional survey. Therefore, a corresponding cohort study with follow-up data should be conducted to strengthen the evidence. Second, the findings were based on self-reported information, and there may be subjectivity derived from this approach in this study.

## Conclusions

This study demonstrated that E2 levels were negatively associated with physical function among the Chinese oldest-old and centenarian women, suggesting that the roles of E2 should be more comprehensively studied in future basic and clinical studies.

## Data Availability

In an attempt to preserve the privacy of individuals, clinical data will not be shared; the data can be available from authors upon request.

## Abbreviations

ADL: Activity of daily living
CHCCS: China Hainan Centenarian Cohort Study
E2: Estradiol
FSH: Follicle-stimulating hormone
LH: Luteinizing hormone
T: Testosterone
